# Young People and the Management of Chronic Illness by Primary Care Pharmacists: A Systematic Review

**DOI:** 10.3390/pharmacy7030089

**Published:** 2019-07-11

**Authors:** Mohammed Almunef, Julie Mason, Chris Curtis, Zahraa Jalal

**Affiliations:** School of Pharmacy, College of Medical and Dental Sciences, University of Birmingham, Birmingham B15 2TT, UK

**Keywords:** Pharmacist, young people and chronic illnesses

## Abstract

Recent evidence has shown that the incidence of long-term illnesses in young people aged 10–24 years is increasing. It is essential to highlight the importance of long-term health conditions in this age group and understand young people’s health needs to be able to improve current support for young people. Pharmacists, as medicine experts, are in a unique position to promote young people’s health. The role of primary care pharmacists in the management of chronic illnesses in young people has not been widely researched. The aim of this review was to explore the current role of primary care pharmacists in the management of chronic illnesses in young people aged 10–24 years. A systematic review was conducted according to the Preferred Reporting Items for Systematic Reviews and Meta-Analyses (PRISMA) statement using Medical Subject Headings (MeSH) and Embase subject headings (Emtree) terms, covering three main themes: Pharmacists, young people and chronic illnesses. Articles were critically appraised using Critical Appraisal Skills Programme (CASP) tools. Eight articles were included in this review. Seven articles included original research studies (one observational study, two surveys, two qualitative interview studies and two interventions). The remaining article was a literature review. All of the articles made reference to community pharmacists, while there was no information about GP pharmacists. Roles that community pharmacists identified as high-priority in their practice when dealing with young people included supporting young people to develop generic healthcare skills, counselling and building trusted relationships directly with young people, helping young people to find credible health information and the provision of specialist services. Community pharmacists feel that they have a role to play in supporting young people with chronic illness and have identified many areas where they can provide services and support.

## 1. Introduction

A UK government survey conducted in 2011 found that approximately 17% of young people aged 10 to 24 years of age have a long-term illness [1]. Common long-term illnesses in this population include asthma, epilepsy and diabetes, and they require long-term treatment with medication to improve disease-related morbidity and mortality. Forty-five million prescriptions were dispensed by community pharmacies for young people in 2017, accounting for approximately 4.1% of all prescriptions and representing about 7.5% of total National Health Service (NHS) prescription spending [2]. These figures are the approximate figures of the total number of prescriptions issued to 0–18-year-olds. 

A review of medicine use in children from the U.S. National Council on Patient Information and Education reported that on average, only 54% adhere to their treatment regimen [3]. Lack of adherence is an issue in patients of all ages, but young people with long-term illnesses face particular challenges. These challenges can be practical, for example, forgetting to fill a repeat prescription or remembering to take medicines, but they are often more complex, with multiple factors influencing medicine-taking behaviours. The needs and understanding of disease and how to manage it change considerably as young people progress through teenage years to adulthood, i.e., as they transition from dependence on carers to self-care. During this transition, young people might find it difficult to make informed and considered decisions about the benefits of taking medicines versus the side-effects sometimes experienced. This could be due to a lack of understanding of the diagnosis and effectiveness of therapy [4]. 

It is not only for reasons of adherence that young people may need additional support from healthcare professionals. They are also at high risk for medication errors, therapeutic misadventures [5] and loss to follow-up in the transition from child to adult services [6]. Therapeutic misadventure can be described as an adverse event or injury caused by medical management rather than underlying disease [7]. In 2007, there were over 86,000 reported adverse events within the NHS [7]. Evidence has shown that medication errors in children occur at similar rates to adults but have three times the potential to cause harm [8].

Recent evidence has shown that the incidence of long-term illnesses in young people aged 10–24 years is increasing [9]. It is essential to highlight the importance of long-term health conditions in this age group and understand young people’s health needs to be able to improve current support for young people. Pharmacists working in the community are easily accessible without an appointment. They are often situated in high streets, neighbourhood centres and supermarkets. For these reasons, pharmacists are in a unique position to promote child and family health through access to needed services and psychosocial support [10]. There is an obvious role for pharmacists in ensuring the safety of medications for young people with long-term conditions through the provision of education to patients/parents [3]. This role was recognised by the 2004 Department of Health and Social Care National Service Framework for Children, Young People and Maternity Services. In addition, community pharmacists could also provide easily accessible continuity of healthcare support during the transition from children’s to adults’ health services. There is a paucity of current available evidence regarding the current role of primary care pharmacists in the management of chronic illnesses in young people aged 10–24 years. The aim of this review was to explore the current role of primary care pharmacists in the management of chronic illnesses in young people aged 10–24 years.

## 2. Materials and Methods

### 2.1. Search Strategy and Data Resources

A systematic review was conducted according to the Preferred Reporting Items for Systematic Reviews and Meta-Analyses (PRISMA) statement by using the four following bibliographic databases: MEDLINE, EMBASE, Cochrane Library and CINAHL. Articles were identified that explored and identified the role of primary care pharmacists in the management of young people with chronic illnesses. Duplicate papers were removed if they were found in different databases. A hand search for relevant references in the various retrieved studies was also undertaken. The search was mainly based on three terms, namely, pharmacist, young people and chronic illnesses. Medical Subject Heading (MeSH) terms and Embase subject headings (Emtree) terms were used in PubMed and Embase, respectively.

### 2.2. Eligibility Criteria 

The scope of the systematic review was limited to research on the role of pharmacists in the management of chronic illnesses in young people in a primary care setting. Studies delivered in primary care or community settings and studies in the English language were included. Articles about the pharmacist’s role in the management of adults (>24 years) and those delivered in a hospital setting were excluded. Articles were excluded if they discussed healthcare services provided by nonpharmacists; if they were on acute illness or disability; or if they were from conferences or were abstracts, letters or case studies. 

### 2.3. Quality Assessment

The quality of included studies was assessed using Critical Appraisal Skills Programme (CASP) checklists [11,12,13]. Due to the methodological heterogeneity of the articles, different types of CASP checklists were used. The articles were classified into three categories: Qualitative studies, cohort studies and literature reviews.

## 3. Results

### 3.1. Data Extraction

**Figure 1:**
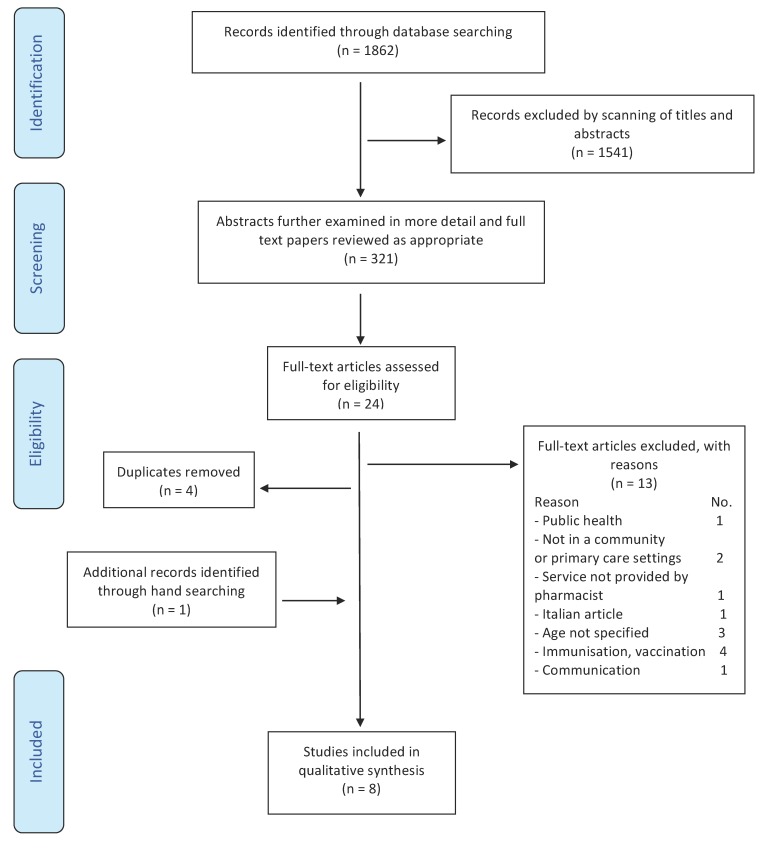
Article selection chart.

The total number of research results after application of the key words was 1862 citations. These citations were extracted from the following databases: MEDLINE (*n* = 690), Cochrane Library (*n* = 38), CINAHL Plus (*n* = 1125), and EMBASE (*n* = 9). After screening the title and abstracts of these articles, 321 articles were found to be relevant. The abstracts and/or the text of these articles were reviewed, and 24 full-text papers were retrieved either electronically or as paper copies for assessment. Seven articles were finally identified. A hand search was completed of the reference lists of the relevant seven articles, and an additional article was added. Therefore, a total of eight articles were considered relevant and were included in this review. Thirteen articles did not match the inclusion criteria for the following reasons: Public health (*n* = 1), not in a community or primary care setting (*n* = 2), service not provided by a pharmacist (*n* = 1), Italian article (*n* = 1), age not specified (*n* = 3), immunization or vaccination (*n* = 4), and communication (*n* = 1). Only full texts of articles were included in this review (see Figure 1).

The eight relevant studies that met the eligibility criteria were conducted in different countries: The UK (three articles), USA (three), Netherlands (one) and Chile (one).

#### 3.1.1. Study Design

Each of the eight articles included in this review had a different study design. Two studies were interviews: One was structured face-to-face interviews with community pharmacy staff members on medication-related problems in adolescents [14], and the other was a qualitative study using semi-structured interviews and thematic analysis with children and parents to explore their perspectives regarding paediatric medication use, knowledge, experiences and pharmacist-provided patient counselling [15]. One study [16] used sequential mixed methods composed of focus groups with community and hospital pharmacists, telephone interviews with pharmacy and rheumatology stakeholders and commissioners and multidisciplinary group discussions to prioritize roles generated by the first two qualitative phases. Benavides et al. [17] conducted a three-month, prospective, cross-sectional study to utilise clinical pharmacists to identify metabolic syndrome in high-risk adolescents. Another study [18] was designed as a semi-structured survey of community pharmacists to explore current practices provided to young people. González-Martin et al., 2003 [19], conducted a paediatric asthma quality-of-life questionnaire (PAQLQ) to measure the impact of a pharmaceutical care programme in two groups of children. An observational study was conducted by Carpenter et al., 2016 [20], to document information about prescriptions that were picked up for children. Finally, one article [3] was a literature review to identify information on managing medicines in children (see Table A1).

#### 3.1.2. Participants/Study Groups

Two of the eight studies that met the eligibility criteria were interventions conducted in young people with specific chronic diseases, asthma [19] and metabolic syndrome [17]. One study was conducted with young people using medications for chronic conditions and their parents [15], and one involved families that picked up prescriptions for young people [20]. Three studies [14,16,18] included community pharmacists exploring the services provided for young people. In Gray et al. [16], rheumatology physicians, nurses and a physiotherapist were also included. Finally, one paper [3] reviewed studies that identified information on managing medicine in children (see Table A1).

#### 3.1.3. Overview of Goals of Studies

In three studies [16,17,19], the general aim of the study was to explore and evaluate the role of pharmacists in the care of young people with chronic illness. The specific objective of González-Martin et al. [19] was to evaluate the impact of pharmaceutical care on children with asthma. In Gray et al. [16], the purpose of the study was to explore the perceived and potential roles of pharmacists in the care of young people with juvenile arthritis. The aim of the work of Benavides et al. [17] was to evaluate the role of a clinical pharmacist in screening adolescents for components of metabolic syndrome. Two studies [14,18] aimed to explore community pharmacists’ perspectives regarding medication-related problems in adolescents: Another objective in Reference [18] was to determine whether community pharmacists undertake medication reviews with children or their carers. One study aimed to explore the perspectives of children and parents regarding paediatric patients’ knowledge and medication use experiences for chronic illnesses, how they want to learn about medicines and perceptions of community pharmacists who provided counselling [15]. The aim of the observational study [20] was to characterise pharmacists’ interactions with children and their caregivers. The aim of the final study in this review [3] was to provide underpinning evidence in the development of advice on managing medicines for children and young people (see Table A1).

#### 3.1.4. Quality of Studies

The overall assessment of the articles was performed by rating the methodological quality. In five articles, most of the criteria were fulfilled [3,15,16,17,19]. In three articles, either not all of the criteria were fulfilled or they were not adequately described, but it was considered that this would have been unlikely to alter the conclusions of these articles [14,18,20]. Therefore, for this review, based on the CASP checklists applied to the eight articles, it is unlikely that different recommendations would have emerged or that different conclusions would have been found from this review.

#### 3.1.5. Setting

In most studies that met the inclusion criteria, the setting was a community pharmacy [14,15,18,20]. In one study, the intervention was in an outpatient paediatric clinic [19], one study was conducted in a paediatric ambulatory clinic [14] and in one study [16] the intervention was convened at targeted paediatric rheumatology centres (see Table A1).

### 3.2. Details of the Extrapolation

All extrapolations were related to the role of pharmacist in the management of chronic illnesses in young people, according to the selection criteria. In this section, interventions are described based on the main component of the study, as some studies consisted of multiple components. Articles were categorised according to the included articles’ descriptions of their interventions and the researchers’ own judgments. Interventions were classified into categories that included pharmacists’ perspectives regarding medication use in adolescent patients, perspectives of adolescents and parents regarding their knowledge and medication use experiences and examples of pharmaceutical care programmes that are provided to young people.

#### 3.2.1. Pharmacists’ Perspectives Regarding Medication Use in Adolescent Patients 

Exploring pharmacists’ perspectives regarding medication use in adolescents was the key objective in three studies [14,16,18]. In the first study [14], there was a structured interview questionnaire that contained questions on medication-related problems in adolescents (especially adherence), the rate of community pharmacist meetings with adolescents (counselling, filling prescriptions) and finally a question about possibilities to improve medication use in young people. The second study [16] included three phases: Focus groups with community and hospital pharmacists, telephone interviews with pharmacy and rheumatology stakeholders and commissioners and multidisciplinary group discussions to prioritize roles generated by the first two qualitative phases. In the first phase, the opinions and experiences of a various group of practicing community and hospital pharmacists about their engagement with adolescents with chronic illness were elicited. This was followed by a second phase to share pharmacists’ ideas about their current and future roles in the support of adolescents with chronic illness, with stakeholders producing a list of roles for prioritisation. The final phase was to encourage pharmacists and rheumatology specialists to prioritise roles to be enhanced or developed. In the third study [18], community pharmacists were asked about their experiences over the last year with young people with chronic illnesses. Many issues were included in the questionnaire, such as administration and obtaining medication, information requests, reported adherence, medication review and adverse effects.

#### 3.2.2. Observation of Community Pharmacists’ Interactions with Young People and Their Carers

In this study [20], the researchers followed an observation guide to record information about prescriptions that were collected for young people. Eight points were documented by research assistants, which included the time of prescription collection, the person who collected the prescription, the person who was counselled by the pharmacist, which pharmacy staff members interacted with the family, the location of pick-up, wait time, how many questions the adolescent or carers asked pharmacy staff and the carers gender. The demographic data of the adolescent and medication information were obtained from the prescription.

#### 3.2.3. Perspective of Adolescents and Parents Regarding Their Knowledge and Medication Use Experiences

One study [15] explored the perspective of adolescents and parents regarding their knowledge and medication use experiences. The intervention used semi-structured interviews to understand the perspectives of adolescents and parents regarding adolescent knowledge and medication use experiences, facilitators of patient counselling and perceptions of community pharmacists. Twenty-minute interviews were conducted by a pharmacy researcher and a research assistant. Interviews were face-to-face for local participants and via telephone for distant participants. The same interview guides were used at both study sites. A participant’s gender, age, race and ethnicity, parent marital status, education and grade level and annual household income were collected.

#### 3.2.4. Pharmaceutical Care Programmes Provided to Young People

Pharmacists provided healthcare services to young people in two studies [17,19]. In the first study [19], young people with moderate asthma who were scheduled for outpatient visits with their internist over a 12-month period were referred for a pharmacist intervention. Participants attending the clinic were allocated into two groups, the intervention group (A) or control group (B). Participants in group A were educated on their disease, self-management, inhalation techniques and pharmacotherapy. Patients in control group (B) were without a pharmaceutical intervention and on their regular treatment for asthma. Spirometry was performed at the beginning of the study and at the completion of nine weeks for both groups. All patients used beta-agonists. To assess the quality of life, a paediatric asthma quality-of-life questionnaire (PAQLQ) was applied to the intervention and control groups at the beginning, after two weeks and after nine weeks. The intervention in the second study [17] aimed to identify metabolic syndrome in high-risk adolescents. At the beginning of the study, a medical history was obtained, a physical examination was performed and a fasting laboratory analysis was carried out. To determine if the adolescent met the criteria for diagnosis, the clinical pharmacist evaluated each component of metabolic syndrome. Then, a summary of the risk factors and treatment recommendations was provided to the paediatrician by the clinical pharmacist. 

#### 3.2.5. Intervention to Improve the Use of Medicines in Young People

A literature review [3] identified interventions on managing medicines in children. The aim was to provide underpinning evidence on the development of advice for managing medicines. This article focused on four areas: Medication review, concordance, enhanced medicine access through community pharmacy services and the use of medicines in schools. Following the eligibility criteria of our review, this report concentrates on “medication review” and “concordance”.

### 3.3. Assessment of Outcomes

#### 3.3.1. Pharmacists’ Perspectives Regarding Medication Use in Adolescent Patients 

This review aimed to explore the role of the primary care pharmacist in the management of chronic illnesses in young people. The high-priority roles for pharmacists that were identified by community pharmacists included developing generic healthcare skills among adolescents, building trusting relationships and direct contact with young people instead of parents, counselling with a concentration on the necessity/benefits of taking medicine, helping adolescents to find credible online health information and use digital media for educational purposes or reminder services, transferring information effectively across care interfaces and developing specialist expertise [14,16]. Around one-half of community pharmacists experienced problems related to medicines and young people. Prescription collection by the parents with or without the adolescent was recognised as an important reason for medication-related problems in adolescents [14]. Pharmacists reported that up to 42.9% of adolescents and/or their carers present to them with nonadherence issues, including stopping medication [14,18].

#### 3.3.2. Perspectives of Adolescents and Parents Regarding Their Knowledge and Medication Use Experiences

Young people and their parents/carers consider community pharmacists to be a resource for information regarding long-term prescribed medication [18]. One study [15] qualitatively explored the perspective of adolescents and parents regarding their knowledge and medication use experiences. Adolescents had minimal knowledge about their medicines, although they were independently managing their medications. The adolescent’s absence during medication collection at pharmacies was a barrier to receiving counselling by pharmacists, as reported by adolescents and parents. Adolescents showed acceptance and were receptive to pharmacists providing education about their medications. The use of educational and interactive technologies for young people consultations were recommended by parents and children.

#### 3.3.3. Pharmaceutical Care Programmes Provided to Young People

Two studies [17,19] evaluated the role of the community pharmacist in providing healthcare services to young people with chronic illnesses (metabolic disease and asthma). Both studies showed significant results in terms of improvement in adolescents’ quality of life and improvement in their knowledge about their disease and its treatment. In the first study [17], a clinical pharmacist provided treatment recommendations for over two-thirds of the young people who participated in the study. The authors concluded that clinical pharmacists can have an effective role in the early identification of specific components of metabolic syndrome. In the other study [19], there was a statistically significant improvement in all of the study’s domains (activities, emotions and symptoms) in the young people who received pharmaceutical care in comparison to those who did not receive it. An improvement in the quality of life of the young people who received pharmacist interventions was reported by the authors.

## 4. Discussion

### 4.1. The Findings

This review was carried out to explore the role of the pharmacist in the management of chronic illnesses in young people in a primary care setting. Eight studies were identified: Interpretation was complex due to the heterogeneity of the studies. The studies were heterogeneous because of the different designs and difference in populations and goals of interventions, and the literature in this area was scarce. The high-priority roles for pharmacists that were identified by community pharmacists included developing generic healthcare skills among adolescents, such as prescription refills and getting free prescriptions; building trusted relationships and direct contact with young people instead of parents; counselling with a concentration on the necessity/benefits of taking medicine; helping adolescents to find credible online health information and use digital media for educational purposes or reminder services; transferring information effectively across care interfaces; and developing specialist expertise [14,16].

It is anticipated that these roles could inform future steps in enhancing the function of community pharmacists in supporting adolescents with chronic diseases [19]. Adolescents are more likely than adults to consider pharmacists as a trustworthy source of information. A survey with a sample size of 4182 was conducted across Germany, Portugal, France and the United Kingdom in 2014 and reported that young people may be more open than adults to utilising a pharmacist as a health information reference [21]. Therefore, resources for providing appropriate care to adolescents, including cognitive and physical development information, should be included in pharmacist training programmes at undergraduate and postgraduate levels. In addition, pharmacists must develop enough specialist knowledge to make certain that medication use is safe for all young people [16]. This can be achieved through the provision of continuous professional development courses and by encouraging pharmacists to attend such training opportunities provided by pharmacy educational and training bodies. 

Pharmacists can play a vital role in identifying patients with medication-related problems. However, there is limited direct contact between community pharmacists and young people. Mostly, parents fill prescriptions at the pharmacy, which might be a cause of medication-related problems among young people being missed [14]. The nonattendance of adolescents during prescription collection may decrease opportunities for communication between pharmacists and adolescents [15]. This has also been reported in other studies: For example, Slack et al., 1995 [22], showed that pharmacists have only a little contact with adolescents. This forms a potential barrier between pharmacists and the adolescent that prevents medication counselling and the provision of instructions related to specific medications, such as the use of subcutaneous injections or inhalers. 

In our review, adolescents were accompanied by their parents during prescription collection almost 32% of the time [15,20]. This finding is similar to that found in previous studies (32% and 50%) [23,24]. Providing counselling by pharmacists to adolescents was very rare even when they were present at the pharmacy [15,20]. Only 2% of adolescents were counselled by their pharmacist [20]. A number of factors may contribute to a low adolescent counselling rate, which have been mentioned in other studies, including a lack of parent interest, a high daily prescription volume, a lack of pharmacist confidence in communicating effectively with adolescents and a lack of time [23,24,25].

It was found that young people aged 10 to 17 years were managing their medicines independently even though they had only a small amount of information about their medications [15]. In contrast, other studies have found that young people with chronic illnesses have expressed worry and fear about self-managing their illnesses [26,27]. Evidence from previous studies has revealed that effective communication with healthcare providers such as pharmacists is deemed to be an approach to improving adolescents’ knowledge and self-management of medications [24,28]. Pharmacists can instruct young people with chronic illnesses on strategies to aid in long-term adherence. This includes ensuring appropriate medication use and showing the proper use of medical devices, for example, metered-dose inhalers [29,30].

Furthermore, the use of social media could be useful in reaching adolescent patients. This tool might enhance adolescents’ medication use behaviour [31]. Young people prefer technology-based education [32,33], and video-based education can improve adolescents’ medication experience [20,29,34]. To encourage patient engagement in disease management, a dashboard with health information was proposed by Judson et al., 2013 [35]. Furthermore, in this review, Koster et al., 2015, [14] suggested the use of new media, text messages, applications and smartphones as solutions to promote counselling and connection with young people.

Community pharmacists have encountered many adolescent-related medication experiences presented to them in their pharmacies. These have included information needs, adherence, formulation issues, medication refills and adverse effects [18]. In a review by Staples and Bravender, 2002 [36], which reviewed studies focusing on medication adherence in adolescents, nonadherence reached a peak during adolescence. The range of adherence found was from 10% to 96%. Nonadherence to medication could be seen in patients as a consequence of the information gap that formed when they commenced their new medication [18]. A previous study in adult patients prescribed a new chronic medication showed that once a patient has taken his/her medication, he/she obtains knowledge about the effect of the drug, and at the same time, new inquiries about the medication arise [37]. In this review, Aston et al., 2017 [18], found that young people ceased taking medication or changed the dose without any advice from the prescriber. This decision was taken either by themselves or through a parent/carer. In Aston et al., 2017 [18], intentional nonadherence was reported by participants more frequently than unintentional nonadherence. More research is needed to explore intended nonadherence in this patient population. 

A study by Koster et al., 2015 [14], reported that low perceived necessity and forgetting to take medication were important reasons for young people not to use medication as prescribed. This result is supported by previous studies [38,39,40]. Solutions that were suggested by community pharmacists to overcome these reasons included specific counselling in the community pharmacy to improve patients’ knowledge and a special focus on the benefits and necessity of medicines [14].

The indication, administration, dose and adverse effects of a medication were the most requested information by patients or their parents/carers [18]. A review by Ryan et al., 2014 [41], which reviewed studies focusing on interventions to improve the safe and effective use of medication by consumers, indicated a scarcity of evidence in adolescents regarding such information. Medication use review services for young people should be extended to provide enhanced contact between young people and community pharmacists to discuss medication issues [18].

### 4.2. Strengths and Limitations

This systematic review had several limitations, which included the short time frame and the heterogeneity of the studies in their methodology and outcomes. In addition, a relatively small number of studies were included, and the systematic review was limited to English language publications only. This study was limited by a focus on chronic conditions and did not consider the array of public health/acute health problems in which pharmacists may be able to support young people. The strengths of the review were that this systematic review applied a vigorous systematic search using diverse databases to identify relevant studies, and it had a focus on pharmacist interventions only.

### 4.3. Implications for Practice

The findings from this review are important for healthcare policymakers and healthcare providers. The provision and evaluation of necessary education and training for community pharmacists is paramount. Training on digital technologies and social media is an area that needs to be focused on to reach young people, and it has the potential to enhance their medication use behaviour. An enhancement of current medicine use review services for young people and their parents would provide an opportunity for community pharmacists to optimise medication use. The education of adolescents with chronic illnesses on strategies to support medication adherence is very important, as the evidence suggests that medication adherence is generally low in adolescents. As there is such a small amount of published work in this area, this highlights the need for additional research to be carried out and published.

## 5. Conclusions

There is a lack of published literature regarding the role of primary care pharmacists in the management of chronic illness in young people. Where pharmacists have managed chronic illnesses in this population, they have been community-based and have had a positive impact on patient outcomes. Pharmacists feel that they have a role to play in supporting young people with chronic illness and have identified many areas where they can provide services and support. However, many pharmacists perceive a fundamental communication barrier that hinders the provision of this support, i.e., lack of access to the patient. Further research is necessary to provide more evidence of the benefit of primary care pharmacists in supporting young people with their medications.

## Appendix A

**Table A1:** Characteristics of the publications included in the review.

Study	Study Aims	Study Type	Setting	Participants
Gray et al. [16], 2017	To explore the perceived and potential roles of pharmacists in the care of young people aged 10–24 years with chronic illness, through the exemplar of juvenile arthritis.	Sequential mixed methods: The first two phases–pharmacist focus groups and stakeholder telephone interviews—were qualitative. The final phase—multidisciplinary discussion groups—was a quantitative approach	Phase 1: Pharmacist focus groups were facilitated by the project manager. Phase 3: Multidisciplinary discussion groups were convened at targeted paediatric rheumatology centres across England and Scotland.	4 focus groups: 9 community pharmacists, 9 hospital Pharmacists.
				15 telephone interviews: 2 pharmacy policymakers, 3 service commissioners, 2 pharmacy staff members, 5 rheumatology professionals, 3 lay advocates.
				3 Discussion groups: 9 community pharmacists, 4 hospital pharmacists, 7 rheumatology physicians, 5 specialist nurses, 1 physiotherapist.
Abraham et al. [15], 2017	To explore the perspectives of children and parents regarding paediatric patients’ knowledge and medication use experiences with chronic illnesses, how they want to learn about medicines and perceptions of community pharmacist-provided counselling.	Qualitative study using semi-structured interviews and thematic analyses.	Three community pharmacies in two eastern states: One in western North Carolina and two in the region of western Pennsylvania.	39 participants: 20 children using medications for chronic illnesses, 19 parents.
Carpenter et al. [20], 2016	To characterize community pharmacists’ interactions with children and their caregivers.	14-day observational study.	Three community pharmacies, one located in a western North Carolina town and two located in western Pennsylvania.	97 families
Benavides et al. [17], 2011	To evaluate the role of a clinical pharmacist in screening children and adolescents for metabolic syndrome.	Three-month, prospective, cross-sectional study.	Paediatric ambulatory clinic located in a community health centre in Texas.	25 participants
González-Martin et al. [19], 2003	To evaluate the impact of a pharmaceutical care program on children with asthma.	Paediatric asthma quality-of-life questionnaire (PAQLQ)in two groups of children:A and B.	Outpatient paediatric clinic of the Catholic University of Chile.	21 recruited children: Group A (11 children), Group B (10 children).
Koster et al. [14], 2015	To explore pharmacy staff’s perspectives regarding medication use behaviour in adolescent patients.	Structured face-to-face interviews.	Community pharmacies in Utrecht.	170 members: 53 pharmacists, 109 technicians, 8 other functions.
Aston et al. [18], 2017	To determine whether community pharmacists undertake medication reviews with children/young people or their parents/carers and to identify the type of medication-related experiences that are presented to community pharmacists when a child/young person is taking long-term prescribed medication.	Semi-structured questionnaire.	England-based community pharmacists.	76 pharmacists
Costello et al. [3], 2004	To provide underpinning evidence in the development of advice on managing medicines for children and young people.	Literature review.	-	31 studies
